# Integrating a High Blood Pressure Advisory Across a Primary Care Network

**DOI:** 10.1001/jamanetworkopen.2025.7313

**Published:** 2025-04-25

**Authors:** Anuradha Phadke, Yingjie Weng, Cati Brown Johnson, Marcy Winget, Megan Mahoney, Christopher Sharp, Amelia Sattler, Shreya Shah, Manisha Desai, Stanley Ng, Jonathan G. Shaw

**Affiliations:** 1 Division of Primary Care and Population Health, Department of Medicine, Stanford University School of Medicine, Palo Alto, California; 2 Quantitative Sciences Unit, Department of Medicine, Stanford University School of Medicine, Palo Alto, California; 3 Department of Family and Community Medicine, University of California San Francisco, San Francisco; 4 University Medical Partners, Livermore, California

## Abstract

**Importance:**

Leveraging technology to prompt team-based care might improve ambulatory hypertension care.

**Objective:**

To assess whether an electronic medical record (EMR) high blood pressure (BP) advisory improves hypertension control.

**Design, Setting, and Participants:**

This quality improvement study assessed hypertension control in patients presenting to primary care office visits from March 2018 to February 2020. Data were included from 28 primary care clinics (8 clinics contributed data toward the primary objective and 28 contributed data toward secondary objectives) in a single academic health system in California before and after intervention and concurrent care team observations and interviews assessing implementation. Data were analyzed from November 2019 to October 2020.

**Intervention:**

An EMR high BP advisory combined with team training, audit, and feedback. EMR entry of elevated BP (systolic BP ≥140 mm Hg or diastolic BP ≥90 mm Hg) prompted an interruptive medical assistant–facing advisory to recheck BP. Persistently elevated BP prompted a second interruptive clinician-facing advisory with order panel link.

**Main Outcomes and Measures:**

The primary outcome was BP lower than 140 mm Hg systolic and lower than 90 mm Hg diastolic during an office visit within 6 months of an initial primary care visit. Secondary outcomes included BP recheck after initial elevated value, antihypertensive medication change, and new hypertension diagnoses. Qualitative outcomes focused on implementation barriers and facilitators.

**Results:**

The primary outcome assessed 2760 control patients and 3018 intervention patients with preexisting hypertension (mean [SD] age, 66.5 [14.4] years; 2847 [49.2%] women, 1746 [30.2%] Asian, 619 [10.7%] Hispanic, and 2407 [41.7%] White). The likelihood of hypertension control increased 18.3% per month on average (odds ratio [OR], 1.18; 95% CI, 1.10-1.27; *P* < .001) in the intervention vs control groups. Modeled rates of adjusted hypertension control over 6 months increased from 82.3% to 92.3% for the intervention cohort and decreased from 71.5% to 70.3% for the control (preintervention) cohort. BP recheck rate increased (from 37.6% to 77.9%; OR, 4.76; 95% CI, 4.45-5.10; *P* < .001), while ordered antihypertensive medications was unchanged. New hypertension diagnosis increased from 12.1% to 20.6% (OR, 1.34; 95% CI, 1.13-1.58; *P* = .01). In interviews of 34 care team members (clinicians, medical assistants, and managers) from 6 clinics, implementation barriers included competing priorities and time for BP rechecks, order panel complexity, and mixed clinician engagement; facilitators included intervention visibility, EMR integration, and team-based approach.

**Conclusions and Relevance:**

This quality improvement study of an EMR high BP advisory intervention found significantly improved primary care hypertension control and diagnosis due to the combination of team-based care and technology.

## Key Points

**Question:**

How was a primary care electronic medical record high blood pressure advisory intervention associated with clinical care and care team experience?

**Findings:**

In this quality improvement study of 14 367 patients from 28 clinics, new hypertension diagnosis increased by 8.5%, and the likelihood of hypertension control (in 5778 patients from 8 clinics) increased a mean of 18% per month. Staff engaged in high rates of blood pressure recheck and reported positive experiences, while clinicians had mixed experiences.

**Meaning:**

These findings suggest that by combining technology and team-based care, a primary care high blood pressure advisory may improve adult hypertension diagnosis and control.

## Teaser

This quality improvement study evaluates whether an electronic medical record high blood pressure advisory was associated with improved hypertension control in an academic health system.

## Introduction

Hypertension is the second most common preventable risk factor for death from any cause.^1^ Health care organizations should play a major role in addressing this condition, yet hypertension is often undiagnosed and undercontrolled in ambulatory care populations.^2,3^

In 2017, our organization formed a primary care team to improve a hypertension control quality metric wherein hypertension control equaled the percentage of patients with a diagnosis of hypertension whose latest office blood pressure (BP) value was less than 140/90 mm Hg and within the last 12 months.^4^ We found uncontrolled or missing values in over one-third of patients, suggesting room to improve management and follow-up.

Inspired by other health systems, we sought to address our gap in hypertension control through an electronic medical record (EMR) high BP and team-based care advisory intervention.^5,6,7^ In this type I hybrid effectiveness-implementation trial, we assessed whether an EMR high BP advisory intervention improved hypertension control (primary objective) and diagnosis, contributors to improved control (BP recheck or medication change), and integration barriers and facilitators.^8^

## Methods

This quality improvement study occurred at Stanford Medicine’s academic primary care network in California. Following a 6-week pilot at a single academic clinic in April and May 2019, 27 additional clinics (13 academic-based and 14 community-affiliated clinics) launched the intervention between May 2019 and January 2020 using a nonrandomized interrupted time series. eFigure 1 in Supplement 1 illustrates the implementation timeline and clinic characteristics. The Stanford University institutional review board designated the study as human participants research exempt.^9^ We followed the Standards for Quality Improvement Reporting Excellence (SQUIRE) reporting guidelines for quality improvement studies in preparing this report.^10^

### Study Design

We used a mixed-methods approach to address our goals: a quantitative design to assess hypertension metrics before and after intervention leveraging concurrent care team observations and interviews to assess implementation. Quantitative evaluation examined data from the preintervention period (from April 2018 to February 2019) and the postintervention period (from April 2019 to February 2020). Due to the COVID-19 pandemic and the rapid shift to virtual care, our study closed early in March 2020. Consequently, only clinics with 6 months of postimplementation data (8 of 28 clinics) contributed to evaluation of the primary objective. All 28 clinics contributed data toward addressing the secondary quantitative objectives. Qualitative evaluation included ethnographic site visits, interviews, and an open-ended questionnaire assessing implementation, conducted by 2 trained qualitative researchers (including C.B.J.) at 6 clinics.

### Study Population

Our study focused on 2 populations. The population for the primary objective was composed of patients with a preexisting diagnosis of hypertension in 8 clinics with a primary care office visit during the study period. Preexisting hypertension was defined by EMR *International Statistical Classification of Diseases and Related Health Problems, Tenth Revision* codes entered in visit diagnoses or antihypertensive medication record in the year before the first primary care visit during the study period (eAppendix in Supplement 1). Consistent with the Medicare Electronic Clinical Quality Measures, we excluded patients with end stage kidney disease, kidney transplant, pregnancy, or hospice enrollment.^4^ The study population for the secondary objective of hypertension diagnosis included all patients without a preexisting diagnosis of hypertension across 28 clinics.

### Intervention

Table 1 details intervention components. Intervention start dates by clinic were staggered based on study design and clinic operational readiness (eFigure 1 in Supplement 1).

**Table 1.** Intervention Components

Component	Recipient	Description
EMR advisory element 1	Medical assistant	Interruptive advisory triggered upon entry of BP reading ≥140 mm Hg systolic or ≥90 mm Hg diastolic in the EMR vitals field Text guidance reads: “BP recheck after two minutes using optimal technique Optimal technique includes: Patient sitting with back supported and feet on the floor for >5 min Arm is bare, relaxed and supported, resting at the level of the heart with palm up Use correct size blood pressure cuff Encourage relaxation breathing” Single option to click “OK” to silence advisory
EMR advisory element 2	Medical assistant	Noninterruptive advisory triggered subsequently, within the rooming navigator of the EMR, prompts: “Please enter second blood pressure reading”
EMR advisory	Clinician	Interruptive advisory triggered upon EMR open Notifies the clinician: “Your patient’s most recent BP reading was elevated” Selection of advisory “Accept” button opens a high blood pressure order panel Alternate selection is “Dismiss”
EMR order panel	Clinician	Clinician hypertension order panel components included: “Hypertension Care Recommended Orders: Referral to pharmacy Referral to clinical nutrition Order of patient portal blood pressure tracker Associated diagnoses - hypertension diagnosis (single response): Essential hypertension Elevated BP without diagnosis of hypertension Recommended Follow-up – appointment options (single response): Schedule with provider in 2-4 weeks Schedule televisit in 2-4 weeks Schedule with medical assistant for BP check in 2-4 weeks After Visit Summary (AVS) documentation: Hypertension general info (English) Hypertension general info (Spanish) Home BP monitoring (English)”
Training materials	Medical assistant	Written and video materials orienting medical assistants to EMR advisory and reviewing proper blood pressure check procedure
Training materials	Clinician	Written and video training materials orienting clinicians to advisory and clinician order panel
Advisory audit report	Clinic manager	Report emailed weekly to clinic managers indicating percentage of times each medical assistant performed BP recheck upon EMR advisory element 1 being triggered Includes performance targets and management suggestions for below target performance
BP check procedure placard	Medical assistant or clinician or patient	Laminated copy of infographic created by the American Heart Association and American Medication Associated entitled “7 Tips to Get an Accurate Blood Pressure Reading”^11^ Copies provided to clinic leaders to post in patient office rooms
Training checklist	Clinic medical director and manager	Checklist provided to clinic leaders encouraging use of above medical assistant and clinician training materials and clinic placards

Abbreviations: BP, blood pressure; EMR, electronic medical record.

### Outcomes

#### Primary Outcome

**Figure 1.**
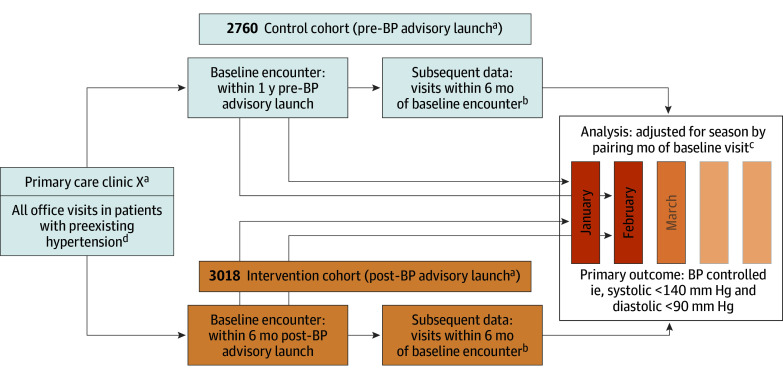
Definition of Analytic Cohort for Primary Outcome in Clinics Receiving the Blood Pressure (BP) Advisory Intervention ^a^In this variant of interrupted time series, the date of the intervention launch varied by primary care clinic site. ^b^Follow-up visits had different cadence and frequency by patient, allowing for varying follow-up schedules and practices. ^c^Primary analysis was also adjusted for patient age, gender, primary insurance type, Charlson comorbidity index, and clinician type; random effects was used to account for correlation of observations over time within a patient and across patients from the same clinic. ^d^Hypertension diagnosis recorded or antihypertensive prescribed within 1 year preceding the baseline encounter.

The primary outcome was BP control, modeled after the National Committee on Quality Assurance 2018 hypertension control quality metric and achieved if the systolic measure was less than 140 mm Hg and the diastolic measure was less than 90 mm Hg.^4^ In cases where multiple BP values were recorded for the same patient on the same day, the lowest systolic and lowest diastolic values were considered the values of record, consistent with the quality metric definition. As shown in Figure 1, this outcome was measured for each participant at multiple and variable visit time points from and up to 6 months after the initial visit. In each clinic, we identified a control cohort of patient visits within the preceding year’s coinciding months to match the same season as the intervention cohort.

Preintervention follow-up time was censored at the time of intervention launch and postintervention follow-up time was censored at administrative study closeout. Only patients with a complete 6-month follow-up window were included in the analysis set of the primary outcome.

#### Secondary Quantitative Outcomes

Our secondary outcomes did not require a 6-month follow-up window. They included the proportion of patients with new hypertension diagnoses within 1 month of clinic encounter (on a patient level), BP recheck rates (on a visit level), and antihypertensive medication prescribing (on a visit level).

Secondary outcomes within the relevant eligible population were defined as follows. First, a new diagnosis of hypertension, defined as a new diagnosis code entered within 1 month of a primary care visit among patients without preexisting hypertension (no hypertension medications or diagnosis in the year before the visit). Second, a BP recheck after an initially elevated reading, defined as a second BP value on a given day among all patients (those with and without preexisting hypertension). Third, antihypertensive medication order during a primary care visit, defined as number of medications or classes of medications ordered per visit (among patients with preexisting hypertension who had antihypertensive medication orders placed during the visit).

We obtained all quantitative data from the Stanford Research Repository, a data warehouse that contains Stanford Health Care EMR data for scholarship.^12^ Qualitative outcomes described clinical care team experience and feasibility.

### Statistical Analysis

Using descriptive statistics (Table 2), we compared the distributions of the patients with hypertension in the control and intervention cohorts. We assessed the patient demographic and clinical characteristics (patient age, gender, race and ethnicity, primary insurance, BMI, key comorbidities, and Charlson comorbidity index), primary care clinician characteristics (attending or resident physician), clinic site level characteristics (community/employer-based, on/off-campus). Race and ethnicity were assessed because of published data demonstrating disparities in US hypertension control rates by race and ethnicity.^3^ We report differences between cohorts using standardized mean difference (SMD.)^13,14^

**Table 2.** Characteristics of the Control and Intervention Patient Cohorts for the Primary Outcome of Hypertension Control Among Patients With Preexisting Hypertension

Characteristic	Patients, No. (%)		SMD^a^
	Control (n = 2760)	Intervention (n = 3018)	
Age, mean (SD)	66.69 (14.19)	66.39 (14.50)	0.021
Gender			
Female	1368 (49.6)	1479 (49.0)	0.028
Male	1392 (50.4)	1538 (51.0)	
Unknown	0	1 (<.01)	
Race and ethnicity			
Hispanic	296 (10.7)	323 (10.7)	0.048
Non-Hispanic Asian	821 (29.7)	925 (30.6)	
Non-Hispanic Black	150 (5.4)	163 (5.4)	
Non-Hispanic White	1164 (42.2)	1243 (41.2)	
Other non-Hispanic^b^	279 (10.1)	292 (9.7)	
Unknown	50 (1.8)	72 (2.4)	
Primary insurance			
Private	1342 (48.6)	1559 (51.7)	0.087
Medicare	1385 (50.2)	1407 (46.6)	
Medi-Cal	23 (0.8)	29 (1.0)	
Self-pay or missing	10 (0.4)	23 (0.8)	
Clinical characteristics			
BMI, mean (SD)^c^	29.19 (7.05)	28.76 (6.69)	0.063
Charlson Comorbidity Index, mean (SD)	1.27 (1.78)	1.29 (1.78)	0.008
Key comorbidity of interest			
Diabetes	736 (26.7)	790 (26.2)	0.011
Diabetes without complications	328 (11.9)	393 (13.0)	0.034
Pulmonary	408 (14.8)	471 (15.6)	0.023
Cancer	367 (13.3)	367 (12.2)	0.034
Kidney diagnosis	355 (12.9)	410 (13.6)	0.021
CHF	279 (10.1)	337 (11.2)	0.034
Liver disease	248 (9.0)	250 (8.3)	0.025
Stroke	222 (8.0)	210 (7.0)	0.041
PVD	202 (7.3)	251 (8.3)	0.037
Medical system characteristics			
Clinics			
On campus academic primary care clinic	1442 (52.2)	1373 (45.5)	0.189
Off campus academic primary care, clinic 1	481 (17.4)	659 (21.8)	
Internal medicine resident continuity, clinic	321 (11.6)	407 (13.5)	
Off campus academic primary care, clinic 2	273 (9.9)	239 (7.9)	
Community-based clinic	161 (5.8)	244 (8.1)	
Employer-based clinic 1	79 (2.9)	88 (2.9)	
Employer-based clinic 2	1 (0.0)	5 (0.2)	
Employer-based clinic 3	2 (0.1)	3 (0.1)	
Clinician type			
Physician	2444 (88.6)	2614 (86.6)	0.059
Trainee (fellow/resident)	316 (11.4)	404 (13.4)	

Abbreviations: BMI, body mass index; CHF, congestive heart failure; PVD, peripheral vascular disease; SMD, standardized mean difference.

^a^ Cohen suggested the magnitude of effect is small if SMD = 0.2, medium if SMD = 0.5, and large if SMD = 0.8.^17^

^b^ Other refers to American Indian, Pacific Islander, or all patients who self-reported their race as other, which is a demographic option within Stanford’s electronic medical record.

^c^ BMI is calculated as weight in kilograms divided by height in meters squared.

We used generalized linear mixed effect models to estimate the overall outcomes of the intervention using the mean difference in monthly changes of BP control rates between the control and intervention cohort using the predicted population margins (with EMMEANS package in R). We conducted both unadjusted and adjusted models and considered the adjusted models our primary analysis. Random effects accounted for correlation of observations over time within a patient and across patients from the same clinic. The fixed effects included: (1) a 0 or 1 variable to indicate whether the BP measure was taken during intervention (1) or not (0); (2) number of months since the index visit; and (3) the interaction of the 2, where the latter is the primary parameter of interest. Our primary models included the following prespecified potential confounders: seasonal effect (month of the year of the initial visit), and the patient-, clinician- and clinic-level characteristics shown in Table 1, including patient age, gender, primary insurance type, Charlson comorbidity index, clinician type at each visit (time-varying), and whether BP is rechecked at each visit (time-varying). We also conducted several sensitivity analyses (detailed in eFigure 2 in Supplement 1) and a secondary analysis modeling the BP as continuous variables in our primary outcome cohort, using linear mixed-effect models with Gaussian distribution (eFigure 3 in Supplement 1).

Similar analyses were run for the secondary outcomes. Analysis of new case recognition was on the patient level with only the initial primary care visits of the incident cases considered, whereas analysis of BP recheck and antihypertensive medication prescribing was performed on the visit level inclusive of all visits. Specifically, we used generalized mixed models (GMMs) to estimate the odds of: (1) a new diagnosis of hypertension in primary care before and after implementing the intervention; (2) medical assistants (MAs) performing a BP recheck; (3) medication change occurring in primary care visits.

Qualitative data were analyzed to capture implementation barriers and facilitators in integrating the high BP advisory and hypertension management into team-based care. Site visit observations and interviews from a varied subset of participating clinics focused on stakeholder experience, implementation feasibility, implementation lessons learned, and potential for maintenance and sustainability. The qualitative data were collected as part of a broader evaluation of hypertension care that contemporaneously evaluated both this intervention and other hypertension-related team-based care across our health system. Here we report only the thematic implementation findings specific to the EMR advisory intervention, as relevant to the quantitative effectiveness findings.

We applied rapid analysis methods to site visits and interviews to provide timely feedback to implementers. Specifically, 2 trained qualitative researchers (including C.B.J.) conducted site visits together, allowing for increased validity of multiple observers. Per the Stanford Lightning Report method, we conducted multiple researcher debriefs daily.^16^ During debriefing and further analysis, disagreement in interpretation was intentionally forefronted, as considering minority opinions in team analysis has been shown to produce better results.^16^ Lightning Reports were shared with quality and clinical leaders and reported at the clinic level to facilitate quality improvement. Quantitative analysis was completed without knowledge of the qualitative findings. Qualitative researchers received quantitative results after conducting site visits but before completing final data analysis.

All hypothesis tests were 2-sided and conducted at the .05 level of significance. All data cleaning was conducted in SAS software version 9.3 (SAS Institute) and statistical analysis was performed in R version 3.4.1 (R Project for Statistical Computing).^15^ Data were analyzed from November 2019 to October 2020 .

## Results

### Primary Outcome: Increase in Hypertension Control in 8 Clinics

Preintervention control (2760 patients) and intervention (3018 patients) patients from 8 clinics were a mean [SD] age of 66.5 [14.4] years, and 2847 [49.2%] were women (Table 2). Characteristics did not differ meaningfully between the control and intervention cohorts. The most common comorbidity was diabetes, followed by pulmonary disease, kidney disease, cancer, and congestive heart failure. A total of 1385 patients (50%) in the control and 1407 patients (47%) in the intervention cohort had Medicare insurance, with the remainder predominantly privately insured; only 1% (23 control and 29 intervention patients) had Medicaid insurance.

**Figure 2.**
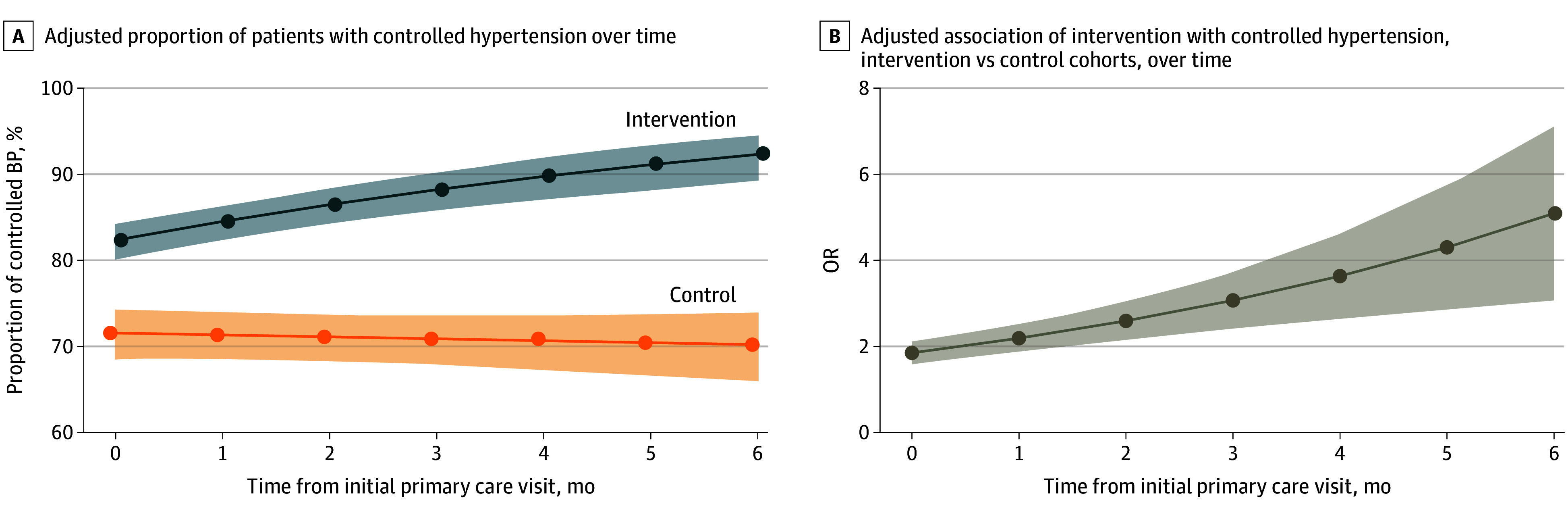
Adjusted Proportion of Patients With Controlled Blood Pressure (BP) Over Time OR indicates odds ratio.

Figure 2 compares hypertension control in each cohort over time. The likelihood of hypertension control increased by 18.3% per month on average (odds ratio [OR], 1.18; 95% CI, 1.10-1.27; *P* < .001) in the intervention vs control cohort. Compared with baseline, modeled rates of hypertension control at 6 months increased from 82.3% to 92.3% for the intervention cohort vs a slight drop from 71.5% to 70.3% for the control cohort.

Results of the sensitivity analyses (eFigure 2 in Supplement 1) were consistent with our primary outcome analysis and demonstrated that the baseline difference between the control and intervention cohorts appears to be due to the lowering of BP values that comes with adding BP recheck to the workflow—that is, if we use only the first measured BP in each cohort (thus ignoring data from repeat measurements inherent in the intervention), the baseline prevalence of controlled hypertension would no longer be observed as differing between cohorts. In the secondary analysis assessing BP changes as a continuous variable (eFigure 3 in Supplement 1), we found a mean reduction of 2.32 (95% CI, −3.09 to −1.55; *P* < .001) and 1.08 (95% CI, −1.63 to −0.53; *P* < .001) mm Hg in systolic and diastolic BP, respectively, at the individual patient level over the 6-month intervention.

### Secondary Outcomes: Increase in BP Recheck and New Hypertension Diagnosis Without Change in Medication Orders

Table 3 shows secondary outcome results. BP recheck after initial elevated BP increased markedly (OR, 4.76; 95% CI, 4.45 to 5.10; *P* < .001) from 37.6% preintervention to 77.9% in postintervention visits. New hypertension diagnosis increased moderately (OR, 1.34; 95% CI, 1.13 to 1.58; *P* = .01). Preintervention, 1423 of 11 806 patients (12%) were newly diagnosed with hypertension as compared with 715 of 3466 patients (20.6%) postintervention. Among visits where antihypertensive medication orders were placed, the mean number of antihypertensive medications or classes of medication per visit did not change.

**Table 3.** Secondary Quantitative Outcomes by Intervention Status Across 28 Clinics

Secondary outcome	Patients, unadjusted No.		Adjusted, OR (95% CI)
	Control	Intervention	
Blood pressure recheck if elevated			
Primary care office visits with elevated BP reading, No.	35 697	16 709	NA
BP recheck complete, %	37.6	77.9	4.76 (4.45-5.10)
New hypertension diagnosis			
Patients without preexisting hypertension, No.	11 806	3466	NA
Patients with new HTN diagnosis, No. (%)	1423 (12.1)	715 (20.6)	1.34 (1.13-1.58)
Antihypertensive orders during primary care visit, No.			
Primary care office visits where antihypertensive orders were placed	8087	3999	NA
Medications per visit, mean (SD)	1.24 (0.54)	1.24 (0.55)	1.02 (0.97-1.07)
Medication classes per visit, mean (SD)	1.10 (0.30)	1.08 (0.27)	0.97 (0.93-1.02)

Abbreviations: BP, blood pressure; HTN, hypertension; NA, not applicable.

### Qualitative Implementation Evaluation: Barriers and Facilitators

A total of 34 individuals (15 clinicians, 11 MAs, 6 managers, and 2 other team members) from 6 clinics participated in semistructured interviews (1 via asynchronous email questionnaire). Qualitative analysis focused on assessing barriers and facilitators encountered in the implementation of the intervention.

#### Barriers to Intervention Implementation

Care team feedback regarding barriers to BP advisory implementation focused on time and competing priorities, variation in implementation and delivery, complexity of the order panel, and mixed degrees of clinician engagement. Clinic managers noted sustainability concerns regarding time needed for BP recheck; after the rollout, some clinics piloted scheduling patients 10 minutes ahead of the clinician visit to increase previsit time for MAs to manage this and other population health initiatives.

In terms of competing priorities, some clinics inadvertently timed their rollout to coincide with another high-volume population health initiative, seasonal influenza vaccination. Specifically, MAs stressed the importance of clinic management considering competing demands and waiting to implement major initiatives if MA responsibilities or patient volumes could be predicted to be seasonably higher (eg, waiting until after influenza season). Finally, most clinicians noted the advisory’s recommended order panel was cumbersome and did not use it, but rather preferred to follow their own usual practices when ordering individual BP medications or laboratories.

Logistical barriers were observed by evaluators during site visits. Patient rooms were not consistently optimized for best practice BP measurements; some had insufficient space, and others had specialized equipment, such as weighing beds, that did not have arm rests or back support to allow for ideal BP measurements. Use of the EMR’s BP advisory order panel by clinicians was rarely observed, consistent with clinicians’ reports of it being cumbersome. Finally, clinician engagement was mixed, creating barriers in some sites or among subsets of clinicians, who, for instance, regularly let patients leave before BP was rechecked.

#### Facilitators to Intervention Implementation

We observed implementation of the BP intervention across all sites and noted several key facilitators: intervention visibility, EMR integration, perceived clinical benefit, and staff engagement. First, the in-room visibility of the intervention was high, supported by the posting of the American Heart Association infographic regarding proper BP measurement in examination rooms.^18^ Second, EMR integration of the intervention promoted sustainability with easy replication across numerous clinic sites. Also, the perceived clinical benefit of the intervention among staff and clinicians supported its acceptability. This, coupled with MA empowerment to independently perform much of the intervention, promoted successful adoption. Notably, MAs at many sites showed strong ownership of the process, including BP recheck, as noted by clinic managers and physicians.

## Discussion

Our mixed-methods study of a team-based EMR high BP advisory intervention found an increase in hypertension control (18% per month increased likelihood across 8 clinics) and hypertension diagnosis (8% increase across 28 clinics). Improvements were associated with marked increase in BP recheck and no change in mean number of antihypertensive medications ordered, underscoring the critical role of accurate measurement in hypertension care. Notably, this intervention’s success occurred despite barriers including uneven clinician support. This suggests that strong MA engagement coupled with EMR integration can create a successful team-based approach to population health management of hypertension.^19,20,21,22,23,24,25^

Our study findings support and augment the recommended CDC approach to high BP advisories. Consistent with CDC recommendations, we found that employing a high BP advisory improves measurement accuracy and control, largely through leveraging the team-based aspect of primary care.^6,7,26^ Augmenting the CDC’s recommendations, we found expanding the advisory’s use in adult patients without preexisting hypertension can improve diagnosis. Additionally, our qualitative findings underscore the importance that an automated advisory build should respect the 5 rights of clinical decision support to maximize clinician acceptability, especially the right format and right time in workflow.^6,27^ Specifically, the clinician advisory’s presentation upon EMR visit encounter opening and the requirement for clinicians to use the order panel to satisfy the advisory likely contributed to poor clinician engagement. Conversely, higher MA acceptability likely corresponded to higher perceived integration with their existing workflow, with the interruptive advisory occurring immediately upon BP value entry.

Qualitative implementation evaluation found high feasibility and penetration of this EMR-based advisory and observed that MA engagement and perceived clinical benefit were key in facilitating success. This study demonstrates that a paired human-technology intervention focused on team-based care and EMR integration is a fruitful approach to improving population health metrics. As health care continues to integrate more technology, grounding care practices in staff empowerment and thoughtful pairing of technology with human support might both increase success and minimize risk of new technologies and processes increasing clinical burnout.^28,29,30,31,32^

From our study, we recommend quality improvement evaluations use combined approaches—quantitative and qualitative—as assessing quantitative outcomes alone, without an eye to the impact on users, is inadequate.^8,33,34^ The implementation findings of our study highlight the importance of ease of adoption for the care team, from thoughtful launch timing (eg, avoiding influenza season) to adapting order panels to increase clinician use.

### Limitations

Our study has several limitations. First, the nonrandomized design risks confounders not fully accounted for in our variant of an interrupted-time-series design with generalized linear mixed models. Our consistency of findings after multiple adjustments and across sensitivity analysis are reassuring. Second, our primary outcome was assessed over 6 months; a longer period would be needed to determine sustainability. Third, our study does not include home BP values, which are increasingly becoming standard of care.^35^ Fourth, generalizability is mixed: while the sites reflect a diversity of clinic types, the study was conducted within an academic health system serving a high proportion of Medicare and employer-insured patients in California.

## Conclusions

Our quality improvement study of a BP advisory intervention that leveraged the EMR and team-based MA role in measurement found that the paired approach enhanced hypertension diagnosis and control. Our findings have encouraged us to pursue an analogous telemedicine advisory to collect home BP values and flag elevated home BP, and expand the office advisory to cardiology. Our findings of mixed clinician experience combined with failure of the clinician advisory to meet our institutional threshold for interruptive advisories have prompted us to shift the clinician advisory to a noninterruptive format and simplify the associated order panel. Overall, we recommend similar pairing of team-based care and technological intervention in other systems seeking to improve hypertension care in our modern value-based reimbursement era.^21,24,25,36,37^ We believe our experience provides strong evidence of effectiveness and implementation learnings.

## Notes

Supplement 1. **eFigure 1.** Intervention Implementation Calendar by Clinic Site **eFigure 2. **Summary of Sensitivity Analyses **eFigure 3. **Changes of Adjusted Systolic and Diastolic Blood Pressure Over Time Since Initial Primary Care Visit, by Preintervention and Postintervention Patient Cohorts **eAppendix. **Definition of Preexisting Hypertension

Supplement 2.
Data Sharing Statement
