# Supplementary material for: Integrating a High Blood Pressure Advisory Across a Primary Care Network
Source: JAMA Netw Open. 2025 Apr 25;8(4):e257313. doi: 10.1001/jamanetworkopen.2025.7313 (PMC12032570; doi:10.1001/jamanetworkopen.2025.7313)
Supplement: Supplement 1. — eFigure 1. Intervention Implementation Calendar by Clinic Site eFigure 2. Summary of Sensitivity Analyses eFigure 3. Changes of Adjusted Systolic and Diastolic Blood Pressure Over Time Since Initial Primary Care Visit, by Preintervention and Postintervention Patient Cohorts eAppendix. Definition of Preexisting Hypertension [file jamanetwopen-e257313-s001.pdf]

Supplemental Online Content

Phadke A, Weng Y, Johnson CB, et al. Integrating a high blood pressure advisory across a primary care network. *JAMA Netw Open*. 2025;8(4):e257313. doi:10.1001/jamanetworkopen.2025.7313

eFigure 1. Intervention Implementation Calendar by Clinic Site
eFigure 2. Summary of Sensitivity Analyses
eFigure 3. Changes of Adjusted Systolic and Diastolic Blood Pressure Over Time Since Initial Primary Care Visit, by Preintervention and Postintervention Patient Cohorts
eAppendix. Definition of Preexisting Hypertension

This supplemental material has been provided by the authors to give readers additional information about their work.

© 2025 Phadke A et al. *JAMA Network Open*.

eFigure 1. Intervention Implementation Calendar by Clinic Site

		Clinics Included in Primary and Secondary Outcomes					Clinics Included in Secondary Outcomes Only					
No.	Code Name	Apr-19	May-19	Jun-19	Jul-19	Aug-19	Sep-19	Oct-19	Nov-19	Dec-19	Jan-20	Feb-20
1	On Campus Academic Clinic #1	x										
2	Employer Based Clinic #1		x									
3	Off Campus Academic Clinic #1		x									
4	Internal Medicine Resident Continuity Clinic		x									
5	Off Campus Academic Clinic #2			x								
6	Employer Based Clinic #2			x								
7	Employer Based Clinic # 3*			x								
8	On Campus Academic Clinic #2						x					
9	Off Campus Academic Clinic #3						x					
10	Express Care Clinic #1						x					
11	Express Care Clinic #2						x					
12	Senior Care Clinic #1						x					
13	Employer Based Clinic #4			x								
14	Employer Based Clinic #5						x					
15	Community Based Clinic #1					x						
16	Community Based Clinic #2										x	
17	Community Based Clinic #3								x			
18	Community Based Clinic #4											x
19	Community Based Clinic #5						x					
20	Community Based Clinic #6											
21	Community Based Clinic #7						x					
22	Community Based Clinic #8										x	
23	Community Based Clinic #9										x	
24	Community Based Clinic #10											x
25	Community Based Clinic #11											x
26	Community Based Clinic #12											x
27	Community Based Clinic #13											x
28	Community Based Clinic #14											x

* Denotes no primary outcome data for this site as no qualifying patients

© 2025 Phadke A et al. *JAMA Network Open*.

eFigure 2. Summary of Sensitivity Analyses

	**Impact of BPA:** *Adjusted Monthly Hypertension Control Rates in Control (Red) and Intervention (Blue) Patient Cohorts*<br>aDID - measured by adjusted ratio of ORs, [95% CI], p-value
**Primary Analysis**	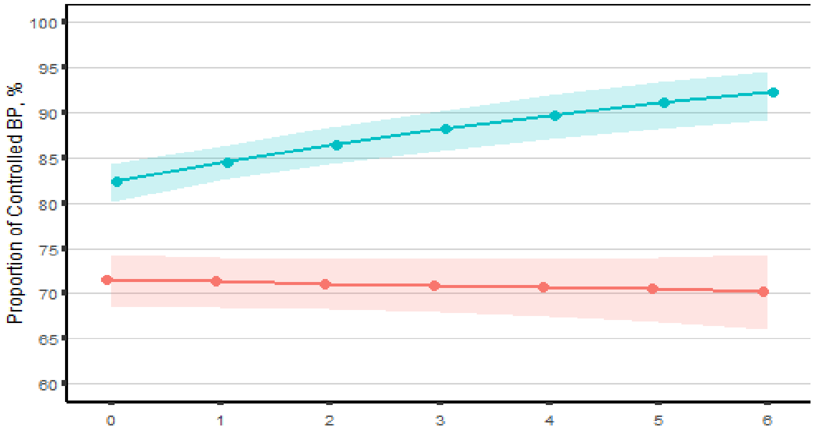 aDID = 1.18 [1.10 to 1.27], p<0.001
**Sensitivity Analysis I:** Outcome using the first measured value at each clinic visit rather than lowest value	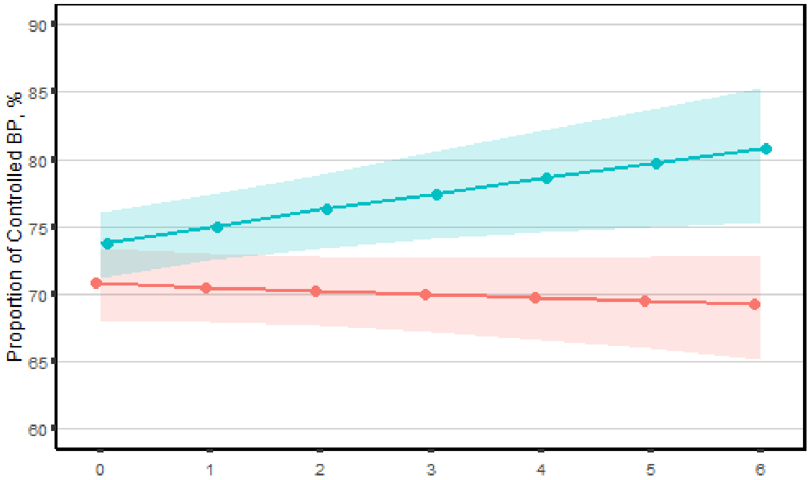 aDID = 1.08 [1.02 to 1.15], p=0.01
**Sensitivity Analysis II:** Outcome including blood pressure measurements at specialty care office visits	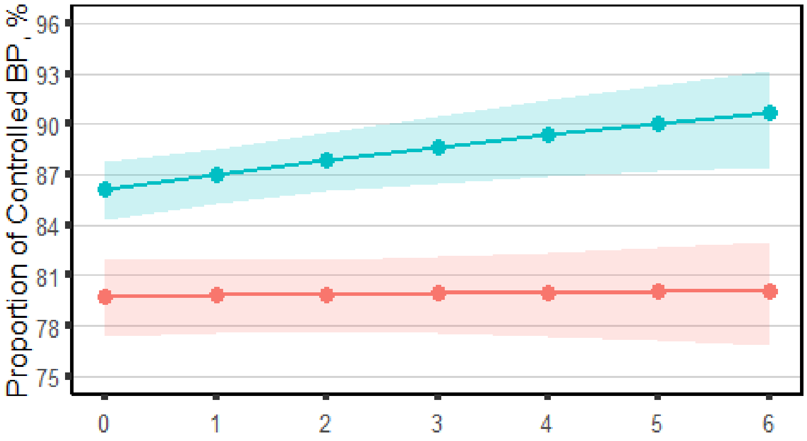 aDID = 1.08 [1.01 to 1.15]. p=0.03

© 2025 Phadke A et al. *JAMA Network Open*.

Sensitivity Analysis III: Assuming a non-linear relationship between the outcome and month from initial visits by including an quadratic term for months from initial visits

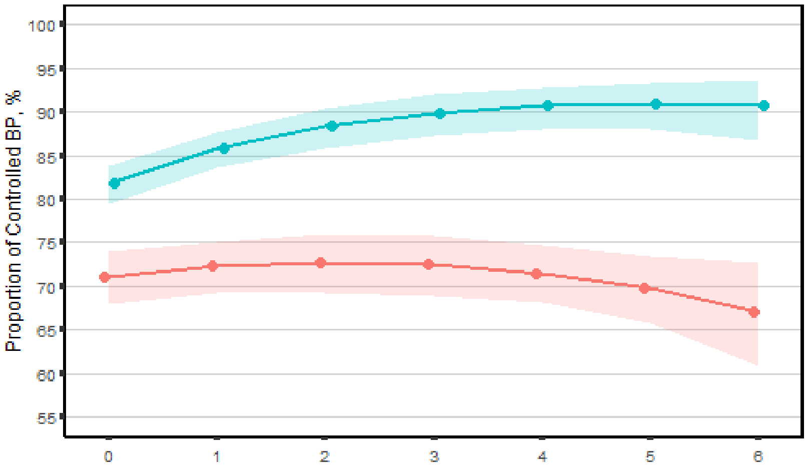

Sensitivity Analysis IV: Temporal trend for pre-intervention patient cohort, compared by the month of patients' initial primary care visits

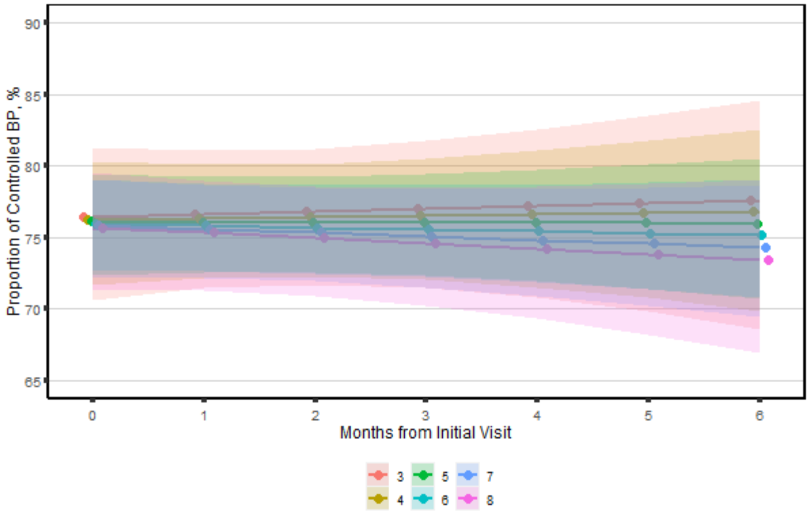

© 2025 Phadke A et al. *JAMA Network Open*.

eFigure 3. Changes of Adjusted Systolic and Diastolic Blood Pressure Over Time Since Initial Primary Care Visit, by Preintervention and Postintervention Patient Cohorts

A. Systolic Blood Pressure

B. Diastolic Blood Pressure

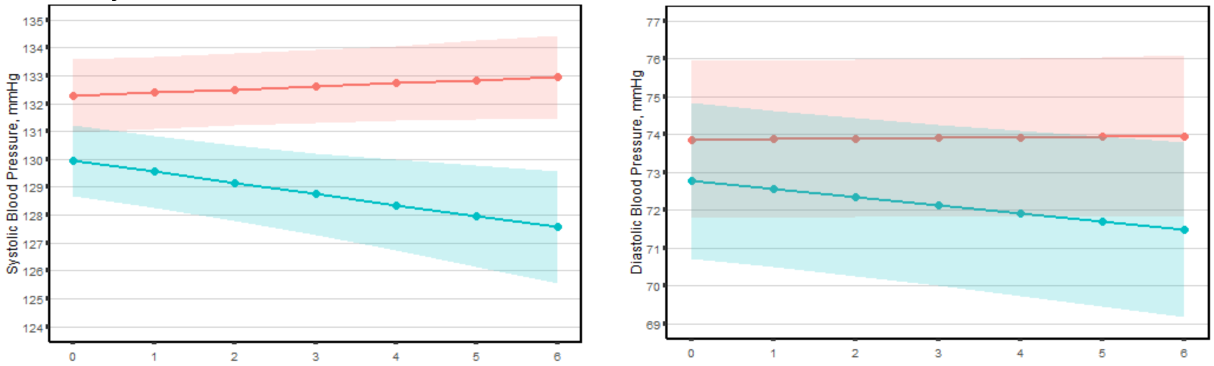

Month from Initial Visit

Adjusted Difference of Monthly Changes in Systolic Blood Pressure (mmHg) Comparing Pre-Intervention and Post-Intervention Patient Cohorts (*aDID*)**:**
-0.51 [-0.84 to -0.19] p=0.002

Adjusted Difference of Monthly Changes in Diastolic Blood Pressure (mmHg) Comparing Pre-Intervention and Post-Intervention Patient Cohorts (*aDID*)**:**
-0.23 [-0.45 to -0.02] p=0.036

Control

Intervention

eAppendix. Definition of Preexisting Hypertension

Patients with pre-existing hypertension were defined as those patients who have an ICD-10 diagnosis code or have any hypertensive medication prescriptions in the past year before their initial primary care visits during the study period.

The hypertension diagnosis code (ICD-10-CM) that were used include the followings: I10, I11.0, I11.9, I12.0, I12.9, I13.0, I13.10, I13.11, I13.2, I15.0, I15.2, I15.8, I15.9. We also included the following classes of hypertensive medication prescriptions: thiazide diuretic (e.g hydrochlorothiazide, chlorthalidone), non-thiazide diuretics (e.g. triamterene, furosemide), aldosterone antagonists (e.g. spironolactone, eplerenone), ACE inhibitors (e.g. lisinopril), angiotensin II receptor blockers (e.g. losartan, valsartan), calcium channel blockers (e.g. amlodipine, felodipine, nifedipine, diltiazem), alpha blockers (e.g. doxazosin, prazosin, terazosin), beta blockers (e.g. metoprolol, carvedilol, labetalol), direct renin inhibitors (e.g. aliskiren), centrally acting agents (e.g. clonidine, guanfacine), vasodilators (e.g. hydralazine, minoxidil), and combination drugs (categories of above are used in combination).

© 2025 Phadke A et al. *JAMA Network Open*.
