# Supplementary material for: Integrating a High Blood Pressure Advisory Across a Primary Care Network
Source: JAMA Netw Open. 2025 Apr 25;8(4):e257313. doi: 10.1001/jamanetworkopen.2025.7313 (PMC12032570; doi:10.1001/jamanetworkopen.2025.7313)
Supplement: Supplement 2. — Data Sharing Statement [file jamanetwopen-e257313-s002.pdf]

Data Sharing Statement

Phadke. Integrating a High Blood Pressure Advisory Across a Primary Care Network. *JAMA Netw Open*. Published April 25, 2025. doi:10.1001/jamanetworkopen.2025.7313

Data
Data available: No
